# Combined Inhibition of EZH2 and FGFR is Synergistic in BAP1-deficient Malignant Mesothelioma

**DOI:** 10.1158/2767-9764.CRC-23-0276

**Published:** 2024-01-03

**Authors:** Jitendra Badhai, Nick Landman, Gaurav Kumar Pandey, Ji-Ying Song, Danielle Hulsman, Oscar Krijgsman, Gayathri Chandrasekaran, Anton Berns, Maarten van Lohuizen

**Affiliations:** 1 Division of Molecular Genetics, The Netherlands Cancer Institute, Plesmanlaan, Amsterdam, the Netherlands.; 2 Oncode Institute, Jaarbeursplein, Utrecht, the Netherlands.; 3 Department of Zoology, Banaras Hindu University, Varanasi, India.; 4 Department of Experimental Animal Pathology, The Netherlands Cancer Institute, Plesmanlaan, Amsterdam, the Netherlands.; 5 Division of Molecular Oncology and Immunology, The Netherlands Cancer Institute, Plesmanlaan, Amsterdam, the Netherlands.

## Abstract

Malignant mesothelioma is a highly aggressive tumor with a survival of only 4–18 months after diagnosis. Treatment options for this disease are limited. Immune checkpoint blockade using ipilimumab and nivolumab has recently been approved as a frontline therapy, but this led to only a small improvement in overall patient survival. As more than half of patients with mesothelioma have alterations in the gene encoding for BAP1 this could be a potential marker for targeted therapies. In this study, we investigated the synergistic potential of combining EZH2 inhibition together with FGFR inhibition for treatment of BAP1-deficient malignancies. The efficacy of the combination was evaluated using human and murine preclinical models of mesothelioma and uveal melanoma *in vitro.* The efficacy of the combination was further validated *in vivo* by using BAP1-deficient mesothelioma xenografts and autochthonous mouse models. *In vitro* data showed sensitivity to the combined inhibition in BAP1-deficient mesothelioma and uveal melanoma tumor cell lines but not for BAP1-proficient subtypes. *In vivo* data showed susceptibility to the combination of BAP1-deficient xenografts and demonstrated an increase of survival in autochthonous models of mesothelioma. These results highlight the potential of this novel drug combination for the treatment of mesothelioma using BAP1 as a biomarker. Given these encouraging preclinical results, it will be important to clinically explore dual EZH2/FGFR inhibition in patients with BAP1-deficient malignant mesothelioma and justify further exploration in other BAP1 loss–associated tumors.

**Significance:**

Despite the recent approval of immunotherapy, malignant mesothelioma has limited treatment options and poor prognosis. Here, we observe that EZH2 inhibitors dramatically enhance the efficacy of FGFR inhibition, sensitising BAP1-mutant mesothelioma and uveal melanoma cells. The striking synergy of EZH2 and FGFR inhibition supports clinical investigations for BAP1-mutant tumors.

## Introduction

Malignant mesothelioma is a treatment-resistant and highly aggressive tumor with a 5-year overall survival rate of only 12%. The majority of cases are causally linked to exposure to asbestos fibres (1, 2). Incidence of mesothelioma has increased worldwide and is predicted to increase fiercely in developing countries (3, 4). Despite recent advances in treatment modalities, including immunotherapy with nivolumab and ipilimumab, the majority of patients die within 1.5 years following diagnosis (5, 6). Many different oncogenic signalling pathways have been implicated in mesothelioma pathogenesis (7, 8). The most prominent pathways reported are PI3K-mTOR-AKT, FGFR, and MAPK pathways (9, 10). In addition, recent literature suggests a critical role of many epigenetic modulators such as KDM6A, SETD2, SETDB6, and EZH2 in malignant mesothelioma development (11).

The genomic landscape of malignant mesothelioma shows frequent inactivation of the *CDKN2AB* locus that encodes for *p16^INK4A^*, *p15^INK4B^*, and *p14^ARF^* cell-cycle inhibitor proteins and the neurofibromatosis type 2 (*NF2*) tumor suppressor gene (12–15). In addition, the gene encoding the BRCA1-associated protein 1 (*BAP1*) is found to be mutated, deleted, or epigenetically silenced in multiple cancers with a prevalence of approximately 60% in the mesothelioma patient population (11, 14, 16–18). BAP1 forms the catalytic core of the polycomb repressive deubiquitinating (PR-DUB) complex, promoting deubiquitination of the repressive H2A-K119ub1 chromatin mark that is deposited by the polycomb repressive complex 1 (PRC1). Polycomb group (PcG) proteins are organized into PRC1 with H2A-K119 ubiquitination activity, and PRC2 with H3K27 methylation activity and are implicated in multiple types of cancers (19–22). EZH2 is a catalytic subunit of the polycomb repressive complex 2 (PRC2) which trimethylates H3K27, consequently leading to gene repression of target genes. Interestingly, we and others have recently found EZH2 to be overexpressed in tumors with loss of BAP1 function, thereby altering the chromatin distribution of PRC1 and PRC2 complexes and leading to a dependency on EZH2 for these tumor cells (23–25).

It has previously been established that inhibition of EZH2 is therapeutically effective in BAP1-deficient mesothelioma in preclinical settings (23, 25). In addition, Quispel-Janssen and colleagues showed that a subgroup of malignant pleural mesothelioma cell lines is sensitive to inhibition of FGFR (7). This subgroup was enriched for BAP1 protein loss which may thus serve as a potential biomarker for a treatment regimen with FGFR inhibitors. However, a phase II clinical trial using the single-agent FGFR inhibitor dovitinib in previously treated patients with mesothelioma showed minimal activity (26). In addition, a phase II clinical trial using the FGFR inhibitor AZD4547 as a second- or third-line treatment against malignant mesothelioma did not demonstrate any benefit in patients who progressed after first-line platinum-based chemotherapy (27). Neither of these studies took BAP1 status of patients into consideration. A recently concluded phase II trial using EZH2 inhibition with tazemetostat in patients with inactivated BAP1 showed modest activity (28). Here, prompted by the minimal efficacy as single agents in clinical trials, we tested whether inhibiting these two targets concurrently has synergistic potential. By combined targeting we were able to demonstrate a significant antitumor effect in both *in vitro* and *in vivo* settings, indicating a potentially promising new treatment modality for BAP1-deficient mesothelioma.

## Materials and Methods

### Cell Culture

Mouse mesothelioma cell lines were previously generated in our laboratory and cultured in DMEM/Nutrient Mixture F-12 (DMEM/F12+Glutamax; Gibco), supplemented with 4 µg/mL hydrocortisone (Sigma), 5 ng/mL murine EFG (Sigma), insulin-transferrin-selenium solution (Gibco), 10% FCS (Capricorn), and 1% penicillin and streptomycin (Gibco; refs. 7, 23). Human mesothelioma cell lines were obtained from the ATCC and were cultured in mammalian cell culture medium as specified above. Uveal melanoma cell lines, also obtained from ATCC, were cultured in either RPMI1640 (Gibco) or DMEM (Gibco) supplemented with 10% or 20% FCS and 1% penicillin/streptomycin. All cell lines were maintained at 37°C in a humidified atmosphere containing 5% carbon dioxide (CO_2_) and were tested for *Mycoplasma* contamination using MycoAlert Mycoplasma detection kit (Lonza). The human cell lines were authenticated using short tandem repeat DNA profiling.

### Knockdown of *Bap1* and *Ezh2* by Inducible Short Hairpin RNA

For *BAP1* knockdown experiments in cell line MP41, we used doxycycline (dox)-inducible FH1-tUTG-RNAi vectors (Taconic Artemis) targeting the following sequence: 5′-GAGUUCAUCUGCACCUUUA-3′. HEK293t cells in 10 cm plates were transduced using 3.5 µg of FH1-tUTG-*BAP1*, 1.1 µg VSV-G, 0.8 µg REV, and 1.6 µg POL. Virus was harvested and used to infect human mesothelioma cell lines. GFP-positive cells were sorted by flow cytometry. To knock down *Ezh2* expression in mesothelioma cells, we used dox-inducible FH1-tUTG-RNAi vectors containing the following targeting sequences: Ezh2-tetKD-A. 5′-GCAAAGCTTGCATTCATTTCA-3′ (29). As a control, we have used Random-tetKD, containing targeting sequence 5′- ATTCTTACGAAACCCTTAG-3′.

### Drug Synergy Assays

Prior to drug synergy assays, optimal seeding density of cell lines was assessed by growth curves. Cells were counted with HyClone Trypan Blue (Cytiva) using a TC20 automated cell counter (Bio-Rad) and alive cells were seeded into 384-well plates in 50 µL of culture medium. Drug compounds, DMSO negative control, or PAO positive control was added after 24 hours using the D300e digital dispenser (TECAN) and cells were grown for 72 hours. Subsequently, cells were incubated for 4 hours with Resazurin (Sigma) and plates were read using an Infinite M1000 pro plate reader (TECAN). Plates were then analyzed using the MacSynergyII program as described by Prichard and colleagues calculating a synergy score for all the combinations (30). Matrix datasets in three replicates were assessed at the 99% confidence level for each experiment. Synergy volume and percent inhibition were calculated and percent inhibition over the predicted effect was plotted in a three-dimensional (3D) plot.

### Colony Formation Assays

Again, prior to colony formation assay optimal seeding densities were determined. Cells were seeded in 6-well culture plates and allowed to adhere overnight. Cells were then cultured in the continuous presence of drug compound(s) or DMSO. After 10 days, plates were fixed using 4% paraformaldehyde (Merck) and stained with 0.1% crystal violet solution (Sigma) in PBS with 10% EtOH. Plates were digitized using ChemiDoc XRS+ (Bio-Rad) and analyzed using the ImageJ plugin “ColonyMeasure” as published by Guzman and colleagues (31). Representative images of three independent experiments are shown.

### RNA Isolation and Gene Expression Analysis

RNA was isolated from tumor cell lines with the Qiagen All Prep DNA/RNA kit. Quantification and quality assessment for RNA were performed with a Bioanalyzer (RNA Integrity Number >6.5; Agilent). Sequencing libraries were constructed with a TruSeq mRNA Library Preparation Kit using poly-A–enriched RNA (Illumina). The Samples were run on a HiSeq 2500 Illumina sequencer generating 65 bp single-end reads. The sequence reads were mapped to the mouse genome (mm10), using TopHat (2.0.12). TopHat was run with default. Reads with mapping quality less than 10 and non-primary alignments were discarded. Remaining reads were counted using HTSeq-count. Statistical analysis of the differential expression of genes was performed using DESeq2 (32). Genes with FDR for differential expression lower than 0.01 were considered significant. Pathway analysis was carried out by the DAVID Kyoto Encyclopedia of Genes and Genomes (KEGG) pathway analysis tools.

### Animal Studies

All animal procedures were performed in accordance with Dutch law and the institutional committees (Animal experimental committee and Animal welfare body) overseeing animal experiments at The Netherlands Cancer Institute, Amsterdam, the Netherlands. Mice were housed under standard feeding, light cycles, and temperature with *ad libitum* access to food and water. All mice were housed in disposable cages in the Laboratory Animal Center of the NKI, minimizing the risk of cross-infection, improving ergonomics, and obviating the need for a robotics infrastructure for cage-washing. The mice were kept under specific pathogen-free conditions.

Maximum tolerability studies were performed in the immune-deficient non–tumor-bearing NOD-Scid IL2Rγnull (NSG) mouse strain and the immune-competent C57BL/6 strain. For 28 days, AZD4547 was administered once daily via oral gavage at a concentration of 7.5 mg/kg in combination with GSK126 given once daily via intraperitoneal injection at a concentration of 40 mg/kg, or tazemetostat administered twice daily via oral gavage at 250 mg/kg. Mice were monitored daily for weight loss, signs of discomfort, abnormal behavior, and death.

To establish xenografts, 5 × 10^6^ mouse mesothelioma-derived cells in 100 µL PBS with 50% Matrigel (Corning) were subcutaneously implanted into the flank of 6 to 10 weeks old NSG mice (Jackson Laboratory). Tumor growth was monitored by slide calliper three times a week (volume = length × width^2^/2). Tumors were allowed to grow to approximately 150 mm^3^ in size before randomization into control and treatment groups. Mice were treated for 21 days. AZD4547 was administered once daily via oral gavage at a concentration of 7.5 mg/kg in combination with GSK126 given once daily via intraperitoneal injection at a concentration of 40 mg/kg, or tazemetostat administered twice daily via oral gavage at 250 mg/kg. Mouse body weight was monitored every day.

Generation of compound knockout mice and induction of autochthonous mesothelioma were executed as described previously (23). Treatments were executed by two independent members of the Intervention Unit of the Netherlands Cancer Institute, the Netherlands. Tumor measurements and health assessments of mice were performed in a blinded manner. Male and female mice were equally distributed over treatment groups with a similar mean weight in each group. Mice receiving different therapies were allowed to be housed in the same cage. Treatment started 4 weeks after tumor induction and continued for 28 days. AZD4547 was administered once daily via oral gavage at a concentration of 5 mg/kg in combination with GSK126 given once daily via intraperitoneal injection at a concentration of 40 mg/kg, or tazemetostat administered twice daily via oral gavage at 250 mg/kg. Mice were monitored daily for weight loss and breathing difficulties. Mice were sacrificed upon signs of illness (breathing abnormalities, kyphosis, weight loss). Kaplan–Meier curves were generated at the end of the experiment.

For histologic analysis, lungs were inflated with formalin or ethanol acetic acid/formalin (EAF). Other tissues harboring tumors were collected separately and also fixed with formalin or EAF for 24 to 48 hours. Fixed tissues were subsequently dehydrated and embedded in paraffin and 2 µm sections were cut and stained for hematoxylin and eosin (H&E).

### Statistical Analysis

All statistical tests were performed using GraphPad Prism v.9 and R. Statistical significance was denoted as *, *P* < 0.05; **, *P* < 0.01; ***, *P* < 0.001; and ****, *P* < 0.0001. The number of independent experiments, samples, and type of statistical test are indicated in the figure legends. No statistical method was used to predetermine the sample size. *In vivo* data were compared by multiple unpaired two-sided Student *t* test when data were normally distributed. Survival analyses were performed by log-rank Mantel–Cox test.

### Data Availability Statement

Data were generated by the authors and are available upon request.

### Ethics Approval

All animal procedures were performed in accordance with Dutch law and the institutional committees (Animal experimental committee and Animal welfare body) overseeing animal experiments at the Netherlands Cancer Institute, Amsterdam, the Netherlands.

## Results

### Combined FGFR and EZH2 Inhibition Reveals Strong Synergy in BAP1-deficient Mesothelioma

**FIGURE 1:**
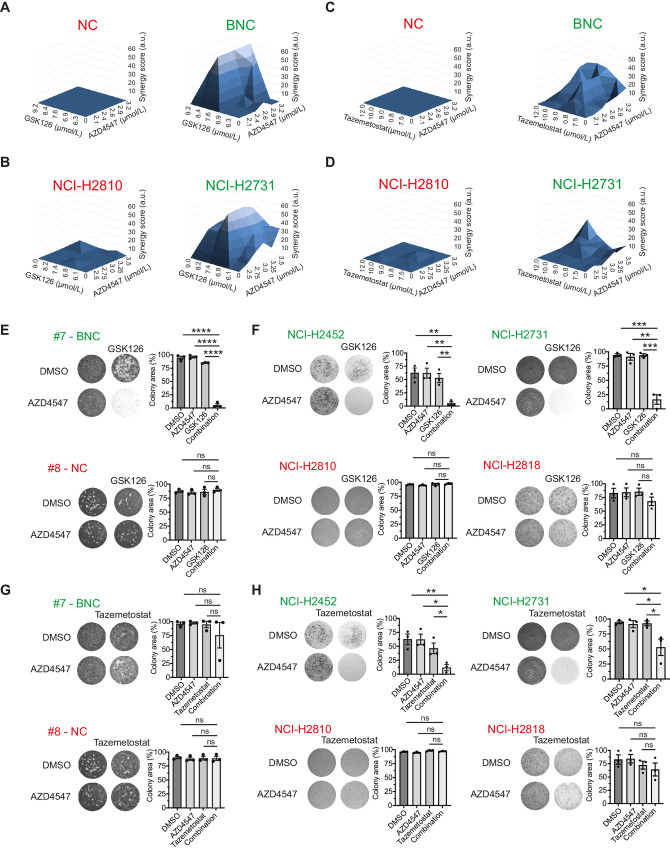
Combined FGFR and EZH2 inhibition reveals strong synergy in BAP1-deficient mesothelioma. **A,** 3D synergy plots visualizing the synergy score for the different concentrations and combinations of FGFR and EZH2 inhibitors in Bap1-proficient (NC) and Bap1-deficient (BNC) mouse mesothelioma cells. Shown are the results of three independent replicates. **B,** 3D synergy plots visualizing the synergy score for the different concentrations and combinations of the inhibitors in BAP1-proficient (NCI-H2810) and BAP1-deficient (NCI-H2731) human mesothelioma cell lines. Shown are the results of three independent replicates. **C,** Similar to the data shown in A, the clinically available inhibitor tazemetostat was used as an EZH2 inhibitor instead of GSK126. **D,** Similar to the data shown in B, the clinically available inhibitor tazemetostat was used as an EZH2 inhibitor instead of GSK126. **E,** Long-term clonogenicity assays of mouse mesothelioma cell lines treated with DMSO, GSK126 (7.5 µmol/L), AZD4547 (0.75 µmol/L), or the combination. **F,** Long-term clonogenicity assays of human mesothelioma cell lines treated with DMSO, GSK126 (7.5 µmol/L), AZD4547 (0.75 µmol/L), or the combination. Representative images are shown from three independent experiments. Quantification data are mean ± SEM, *n* = 3 independent experiments. Representative images are shown from three independent experiments. Quantification data are mean ± SEM, *n* = 3 independent experiments. **G,** Similar to the data shown in E, the clinically available inhibitor tazemetostat (10 µmol/L) was used as an EZH2 inhibitor instead of GSK126. **H,** Similar to the data shown in F, the clinically available inhibitor tazemetostat (10 µmol/L) was used as an EZH2 inhibitor instead of GSK126; images of control wells are duplicated for presentation purposes, *P* values were determined by using two-tailed unpaired Student *t* test; ^∗^, *P* < 0.05; ^∗∗^, *P* < 0.01, ^∗∗∗^, *P* < 0.001; and ^∗∗∗∗^, *P* < 0.0001.

To investigate whether a combined treatment of EZH2 and FGFR inhibition is effective, we made use of mouse mesothelioma cell lines derived from our genetically defined BNC (*Bap1*^−^*^/^*^−^*, Nf2*^−^*^/^*^−^*, Cdkn2ab*^−^*^/^*^−^) and NC (*Nf2*^−^*^/^*^−^*, Cdkn2ab*^−^*^/^*^−^) mice (23). The cell lines used in this study were early passage (within the first 4 to 8 passages) derived from tumors isolated from BNC and NC mice. Cells were treated with varying concentrations of GSK126, AZD4547, and combinations of both. After 72 hours of treatment, cell viability was measured. Potential synergy and drug interactions were quantified using the MacSynergy II algorithm by computing a synergy score. For the combination, we found an overall synergy score >30 in Bap1-deficient mouse mesothelioma cell lines which is generally accepted as highly synergistic potential (Fig. 1A). In contrast, in Bap1-proficient lines, no synergy was observed. To assess the potential clinical value of our finding, we tested a BAP1-deficient and a BAP1-proficient human mesothelioma cell line. Assessing these lines in a similar manner again showed a high synergy score for the BAP1-deficient line but not for the BAP1-proficient human cell line (Fig. 1B). Substituting our EZH2 inhibitor GSK126 with the clinically available tazemetostat (EPZ-6438) showed results consistent with our previous data in both mouse and human cell lines (Fig. 1C and D).

To further validate these findings, we performed long-term low-density clonogenicity experiments in an extended panel of mesothelioma cell lines. These assays were quantified by using the ColonyMeasure tool in ImageJ (31). Here, we observe that the combination is able to nearly completely inhibit cell proliferation in BAP1-deficient settings exclusively whereas single-agent treatment has little effect on cell survival (Fig. 1E and F). Furthermore, we expanded these colony formation assays by using the alternative EZH2 inhibitor, tazemetostat together with AZD4547, also showing significant growth reduction albeit more modest than the combination with GSK126 (Fig. 1G and H). Finally, we performed genetic depletion of EZH2 via inducible short hairpin RNA (shRNA) and show that these cells are more sensitive to FGFR inhibition than shRNA control cells providing evidence that the described sensitivity is not inhibitor specific (Supplementary Fig. S1A and S1B). Taken together, our *in vitro* results show a high synergistic potential for combining FGFR and EZH2 inhibition against BAP1-deficient malignant mesothelioma.

### EZH2 Inhibition Results in Upregulation of FGFR Pathways

**FIGURE 2:**
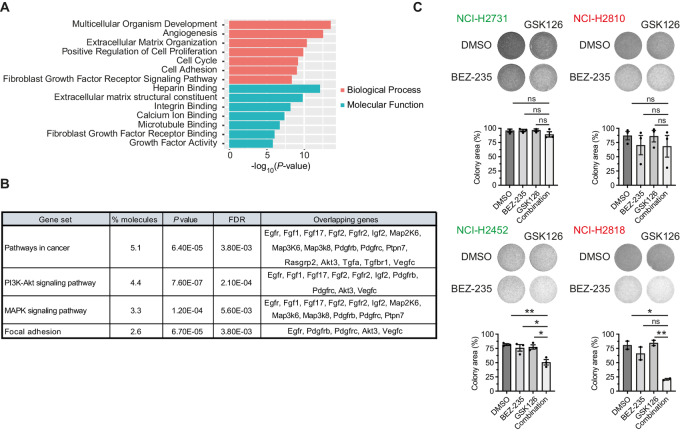
EZH2 inhibition results in upregulation of FGFR pathways. **A,** GO-term analysis on differentially expressed genes between BNC cell lines (*n* = 2) that were treated with GSK126 or untreated. **B,** KEGG pathway enrichment analysis on samples mentioned in A. **C,** Long-term clonogenicity assays of human mesothelioma cell lines treated with DMSO, GSK126 (7.5 µmol/L), BEZ-235 (15 nmol/L), or the combination. Representative images are shown from two independent experiments.

To gain insight in the molecular consequences of EZH2 inhibition and the increased sensitivity to the combination in BAP1 depleted cells, we performed RNA sequencing (RNA-seq) on primary tumor cell lines treated with EZH2 inhibitor. We performed differential mRNA expression analysis between EZH2 inhibitor treated and untreated BNC cell lines. We found 935 genes to be upregulated (*P* < 0.01) in BNC cells upon GSK126 treatment. Gene Ontology (GO)-term analysis on these genes showed a significant enrichment for both FGFR signaling pathway as well as for binding of a number of constituents (Fig. 2A). In addition, KEGG pathway enrichment analysis of genes deregulated in mesothelioma in which *Bap1* was disrupted revealed an overall prevalence of genes implicated in PI3K-Akt and MAPK signalling among others (Fig. 2B). As we found PI3K-Akt signalling pathways to be enriched upon EZH2 inhibition, we checked whether combining EZH2 inhibition with a PI3K inhibitor would also show synergistic effects. Therefore, we performed clonogenicity assays in human mesothelioma cell lines combining GSK126 with BEZ-235 (Dactolisib), an inhibitor of PI3K. This combination was not effective in all the cell lines which could not be attributed to BAP1 status–specific efficacy, unlike the EZH2i + FGFRi combinations (Fig. 2C). These data suggest that the transcriptional upregulation of FGF/FGFR genes upon EZH2 inhibition might partially explain the observed synergy of combined FGFR and EZH2 inhibition in mesothelioma.

### EZH2 Inhibition Together with FGFR Inhibition is Synergistic in Uveal Melanoma

**FIGURE 3:**
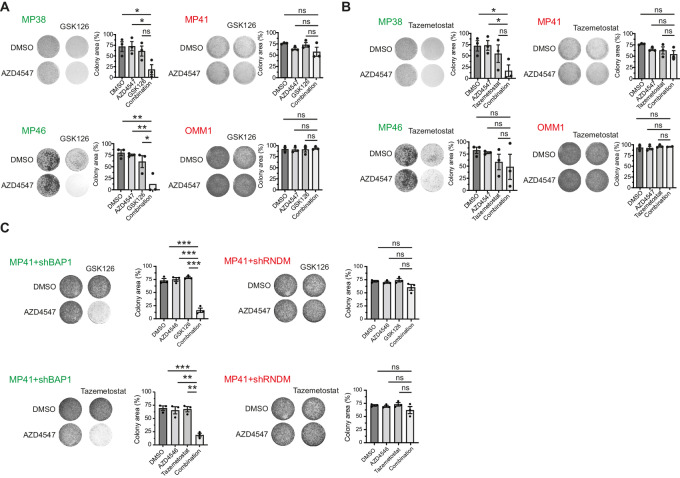
EZH2 inhibition together with FGFR inhibition is synergistic in uveal melanoma. **A,** Long-term clonogenicity assays of human uveal melanoma cell lines treated with DMSO, GSK126 (7.5 µmol/L), AZD4547 (0.75 µmol/L), or the combination. **B,** Similar to the data shown in A, the clinically available inhibitor tazemetostat (10 µmol/L) was used as an EZH2 inhibitor instead of GSK126. **C,** Clonogenicity assays of the uveal melanoma cell line with inducible shRNA against BAP1 or shRANDOM treated with DMSO, GSK126 (7.5 µmol/L), tazemetostat (10 µmol/L), AZD4547 (0.75 µmol/L), or a combination of the EZH2 inhibitor and FGFR inhibitor; Representative images are shown from three independent experiments, images of control wells are duplicated for presentation purposes,. Quantification data are mean ± SEM, *n* = 3 independent experiments, *P* values were determined by using two-tailed unpaired Student *t* test; ^∗^, *P* < 0.05; ^∗∗^, *P* < 0.01; ^∗∗∗^, *P* < 0.001.

As *BAP1* is frequently mutated in patients with uveal melanoma and seems to be highly prevalent in liver metastasis, with a prevalence of approximately 95%, we tested whether this combination is effective against uveal melanoma as well. Therefore, we performed long-term colony formation assays in human uveal melanoma cell lines with different BAP1 status. Cell lines were treated with DMSO, single-agent AZD4547 or GSK126, or its combination. We found that, likewise to mesothelioma, BAP1-mutated uveal melanoma cells are hypersensitive to this drug combination whereas BAP1 wildtype cells are not (Fig. 3A). Replacing GSK126 with tazemetostat yielded similar results (Fig. 3B). In addition, we generated an isogenic variant of MP41 by using an inducible shRNA against *BAP1* (Supplementary Fig. S2). Treatment of this cell line and its BAP1-proficient counterpart show that sensitivity to the combination is dependent on the BAP1 status of the cells (Fig. 3C). These data suggest that the tested combination could also be used as a therapeutic strategy against BAP1-deficient uveal melanoma and potentially to a multitude of *BAP1*-mutated malignancies.

### EZH2 Inhibition Together with FGFR Inhibition is Well Tolerated and Shows Reduced Tumor Growth in Xenograft Models of Mesothelioma

**FIGURE 4:**
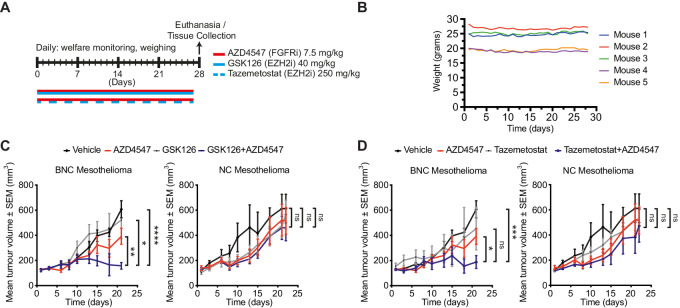
EZH2 inhibition together with FGFR inhibition is well tolerable and shows reduced tumor growth in xenograft models of mesothelioma. **A,** Schematic representation of the treatment schedule of the MTD. NSG mice were treated with a combination of AZD4547 (FGFRi) and GSK126 (EZH2i) or AZD457 and tazemetostat (EZH2i) for 28 consecutive days. **B,** Body weight of individual mice in the treatment group over time. Shown are C57BL/6 mice from the AZD4547/GSK126 cohort. **C,** NSG mice with BNC and NC xenografts were treated with vehicle, AZD4547 (7.5 mg/kg once daily), GSK126 (40 mg/kg once daily), or a combination. Shown is mean tumor volume over time (tumor volume ± SEM; *n* = 8 mice per treatment group). **D,** NSG mice with BNC and NC xenografts were treated with vehicle, AZD4547 (7.5 mg/kg once daily), tazemetostat (250 mg/kg twice daily), or a combination. Shown is mean tumor volume over time (tumor volume ± SEM; *n* = 8 mice per treatment group); *P* values were determined by using two-tailed unpaired Student *t* test; ^∗^, *P* < 0.05; ^∗∗^, *P* < 0.01, ^∗∗∗^, *P* < 0.001; and ^∗∗∗∗^, *P* < 0.0001.

Because of the absence of data on combined toxicity for these drugs, we performed a tolerability study in non–tumor-bearing NSG mice and in immune-competent C57BL/6 to determine the MTD of this combination. On the basis of existing literature and prior experience using these inhibitors as single agents, AZD4547 was administered once per day via oral gavage, GSK126 was administered once per day via intraperitoneal injection, and tazemetostat was administered twice per day via oral gavage. All drugs were administered for a period of 28 days (Fig. 4A). For the duration of the experiment, endpoints of dose-limiting toxicity were weight loss of more than 20%, abnormal behavior, signs of discomfort, and death. The chosen dosages were well tolerated in both immune-competent and immune-deficient mouse models according to body weight and mouse survival (Fig. 4B; Supplementary Fig. S3A–S3C).

To test the effect of combined EZH2/FGFR inhibition *in vivo*, mouse mesothelioma cells that were either Bap1-proficient (NC) or -deficient (BNC) were grafted subcutaneously into the flanks of mice and treated with the drug combination. Mice bearing tumors with a volume of minimally 150 mm^3^ were randomly assigned into Vehicle, GSK126 (40 mg/kg, by daily intraperitoneal injection), AZD4547 (7.5 mg/kg, by oral gavage once daily), or combination cohort and subsequently treated for 21 days. We observed a modest effect on tumor growth by single-agent GSK126 or AZD4547 treatment both in NC and BNC xenograft tumors (Fig. 4C). However, the combination of GSK126 and AZD4547 therapy significantly reduced tumor growth in BNC mice as compared with NC mice. We also treated the xenograft tumors with the EZH2 inhibitor, tazemetostat (250 mg/kg, twice daily by oral gavage). Again, we observed a pronounced tumor reduction in BNC xenograft mice but not in NC mice (Fig. 4D). The results obtained by double agent treatment *in vivo* in xenografts suggests a combination effect by EZH2i/FGFRi treatment and aligns with our *in vitro* findings of increased sensitivity against Bap1-deficient mesothelioma tumor cells.

### EZH2 and FGFR Combination Prolong Survival of Autochthonous BNC Mouse Models

**FIGURE 5:**
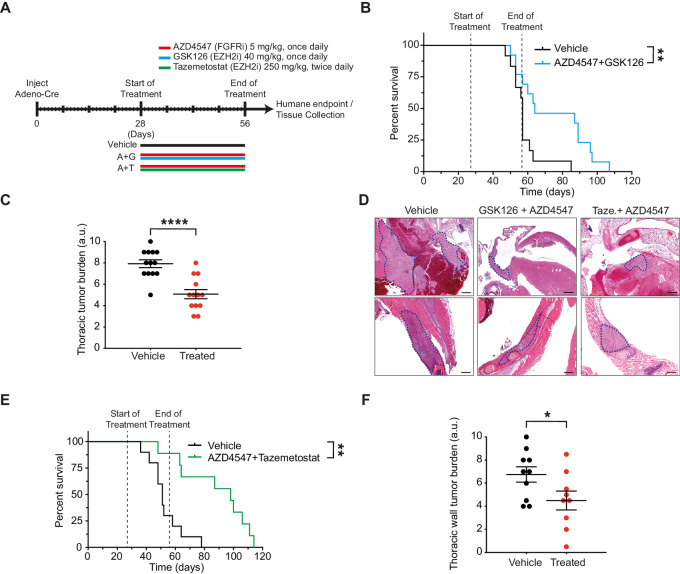
EZH2 and FGFR combination prolong survival of autochthonous BNC mouse models. **A,** Schematic representation of the experimental workflow of the autochthonous *Bap1-*deficient mesothelioma mouse model. **B,** Kaplan–Meier curve comparing the survival of vehicle-treated, and AZD4547/GSK126-treated mice (*n* = 11 mice per treatment group). AZD4547 was administered via oral gavage once daily at 5 mg/kg. GSK126 was administered once daily via intraperitoneal injection at 40 mg/kg. Dashed lines indicate start and end of treatment. *P* value was determined by log-rank (Mantel–Cox) test; **, *P* < 0.01. **C,** Thoracic tumor burden of the mice in the cohorts was analyzed and scored by an experienced animal pathologist. *P* value was determined by two-tailed unpaired Student *t* test; ****, *P* < 0.0001. **D,** Microphotographs of H&E staining showing tumor lesions in myocardium of heart (top) and in thoracic muscle wall (bottom) from treated and vehicle mice. Major parts of the tumor area are highlighted with elliptical boxes. Scale bars: 500 µm. Representative images are shown. **E,** Kaplan–Meier curve comparing the survival of vehicle-treated, and AZD4547/tazemetostat-treated mice (*n* = 11 mice per treatment group). AZD4547 was administered via oral gavage once daily at 5 mg/kg. Tazemetostat was administered twice daily via oral gavage at 250 mg/kg. Dashed lines indicate start and end of treatment. *P* value was determined by log-rank (Mantel–Cox) test; **, *P* < 0.01. **F,** Thoracic wall tumor burden of the mice in the cohorts was analyzed and scored by an experienced animal pathologist. *P* value was determined by two-tailed unpaired Student *t* test; *, *P* < 0.05.

Previously, we have described immune-competent autochthonous BNC and BNC^a^ mouse models that rapidly develops highly aggressive mesothelioma (23). Having shown the potency of the EZH2i + AZD4547 combination treatment compared with the monotherapies in BNC xenografts, we decided to further substantiate our observations in an immunocompetent autochthonous mouse model. A maximum tolerability study with the combination in this model was done prior to the experiment. Tumor development was initiated by conditional deletion of the BNC alleles by injecting Adeno-Cre virus into the mice, followed by therapeutic intervention starting 4 weeks later for 4 weeks. Mice in the combination group were treated with GSK126, 40 mg/kg, by intraperitoneal injection, once daily, and AZD4547, 5 mg/kg, by oral gavage, once daily (Fig. 5A). Mice were monitored until they showed signs of respiratory distress, significant weight loss, or otherwise reached their humane endpoint. Combination treatment in this “worst-case” scenario mouse model significantly increased survival (Fig. 5B). In addition, we analyzed and compared the tumor burden of the treated and non-treated mice. In accordance with the prolonged survival, we found a significantly reduced thoracic tumor burden upon combination treatment (Fig. 5C and D). Using tazemetostat rather than GSK126 in a second autochthonous mouse mesothelioma model (BNC^a^) showed an even bigger increase in median survival (98 vs. 51 days) upon treatment with the combination as compared with the vehicle cohort (Fig. 5E). The thoracic wall tumor burden was significantly reduced for the combination with tazemetostat as compared with vehicle treated mice (Fig. 5F).

Taken together, we were able to show that a combination of FGFR and EZH2 inhibitors is a potentially promising therapeutic strategy for the treatment of BAP1-mutant mesothelioma cells. We showed high synergy in *in vitro* settings and were able to demonstrate a substantial antitumor effect in different *in vivo* settings thereby prolonging mouse survival.

## Discussion

The current standard of care for patients with mesothelioma consists of traditional chemotherapeutic interventions and the recently approved immune checkpoint blockade therapy using nivolumab and ipilimumab. Immunotherapies have fared well in hematologic malignancies and show a lot of promise in several solid tumor types, including mesothelioma; however, there still is a significant fraction of non-responding patients that will benefit from alternative therapies. Therefore, new treatment strategies tailored for molecularly selected patient population is worth further exploring in such difficult to treat malignancies.

BAP1 appears one such biomarker gene which is mutated in a large fraction of mesothelioma and has been used for exploring pharmacologically targetable vulnerabilities for patients lacking BAP1. For instance, a recent study assessed the clinical activity of PARP inhibition using niraparib in patients with advanced tumors likely to harbor mutated BAP1 (33). While clinical benefit was identified in the majority of patients with a mutated BAP1 tumor, the use of niraparib failed to meet the prespecified efficacy threshold of an objective response rate. Also, preclinical reports by us and others have shown that inhibition of EZH2 is therapeutically effective against BAP1-deficient mesothelioma (25). However, our results from mesothelioma mouse models show that tazemetostat may show limited efficacy when used as a single agent (23, 24). Indeed, a recently concluded phase II trial using EZH2 inhibition with tazemetostat in patients with inactivated BAP1 showed only modest activity corroborating our findings in mice (28). In another study that screened for novel vulnerabilities in mesothelioma, inhibition of FGFR was shown to be effective against a subgroup of mesothelioma cell lines enriched for BAP1 alterations (7). Phase II clinical trials using single-agent FGFR inhibition in patients with mesothelioma that were treated previously, however, showed no to minimal activity or benefit (26, 27). Notably, patients in this study were not stratified for their BAP1 status. All these observations underscore that available monotherapies for BAP1-deficient mesotheliomas are suboptimal and therefore exploring drug combinations seems prudent. Moreover, the drugs which have favourable toxicity profiles such as epidrugs could be used as an anchor drug for drug synergy studies.

In this study, we have combined the inhibition of the two previously reported vulnerabilities associated with the loss of *BAP1* in patients with mesothelioma. We were able to demonstrate that using these inhibitors simultaneously leads to superior results over single-agent use in *in vitro* settings. Treating murine and human mesothelioma cell lines with concentrations that have no or little effect on cell viability as single agent led to an almost complete loss of cell survival when combined together, and this effect was exclusively observed in BAP1-deficient cell lines. In addition to the high sensitivity of BAP1-mutant mesothelioma, we show that the treatment of EZH2 inhibitor in conjunction with FGFR inhibitor is effective in uveal melanoma as well. Similar to mesothelioma this effect in uveal melanoma is restricted to BAP1-deficient cell lines. This is particularly interesting as ±95% of metastasized uveal melanoma tumors are BAP1 mutated.

Using multiple preclinical models, we report that the synergistic effect of the combination causes a reduction in tumor growth in xenograft experiments to an extent not observed for single-agent treatments. In addition, in our MTD study, we show that this combination is well tolerable in both immune-deficient and immune-competent mice. Importantly, using our highly aggressive autochthonous models of mouse mesothelioma we show that combined treatment prolongs the median survival and lowers the thoracic tumor burden. Our *in vivo* studies show that the combined treatment with GSK126 and AZD4547, tazemetostat and AZD4547 confers substantial stronger antitumor activity than either inhibitor alone in BAP1-deficient tumors in mice along with strong effect on progression-free survival.

Our observations clearly indicate that BAP1-deficient mesothelioma is sensitive to the combination of EZH2 inhibition and FGFR inhibition, thereby providing a molecularly rationalized approach that should encourage clinical investigation of the combined therapy with EZH2 and FGFR inhibitors in BAP1-deficient cancer. The precise mechanism by which these cells are sensitized to the combination is a limitation of the study and should be investigated further. In view of the clinical availability of the inhibitors used against EZH2 (tazemetostat) and FGFR (AZD4547), this highly synergistic combination is worth testing as a therapeutic regimen against mesothelioma and potentially other BAP1-mutated malignancies as well.

## Supplementary Material

Supplementary Figure S1 Supplementary Figure S1 shows that knock-down of EZH2 sensitises Bap1-deficient mesothelioma cells to FGFR inhibition.

Supplementary Figure S2 Supplementary Figure S2 shows efficient knock-down of BAP1 via inducible shRNA in uveal melanoma.

Supplementary Figure S3 Supplementary Figure S3 shows stable body weight of mice treated with the combination.
