# Supplementary material for: Combined Inhibition of EZH2 and FGFR is Synergistic in BAP1-deficient Malignant Mesothelioma
Source: Cancer Res Commun. 2024 Jan 3;4(1):18–27. doi: 10.1158/2767-9764.CRC-23-0276 (PMC10763530; doi:10.1158/2767-9764.CRC-23-0276)
Supplement: Supplementary Figure S1 — shows that knock-down of EZH2 sensitises Bap1-deficient mesothelioma cells to FGFR inhibition. [file crc-23-0276-s01.pdf]

Supplementary Figure S1

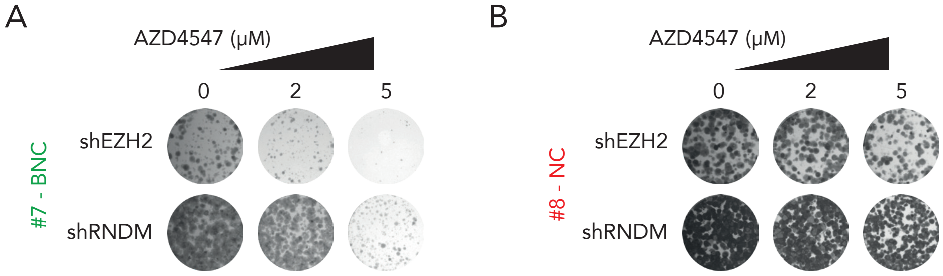

Supplementary Figure S1. EZH2 knock-down sensitizes BAP1-deficient mesothelioma cells to FGFR inhibition. A, Long-term clonogenicity assays of Bap1-deficient mouse mesothelioma cell line (BNC) with an inducible shRNA against EZH2 or shRandom treated with the FGFR inhibitor AZD4547. **B,** Likewise, for the Bap1-proficient cell line (NC).
