# Supplementary material for: Combined Inhibition of EZH2 and FGFR is Synergistic in BAP1-deficient Malignant Mesothelioma
Source: Cancer Res Commun. 2024 Jan 3;4(1):18–27. doi: 10.1158/2767-9764.CRC-23-0276 (PMC10763530; doi:10.1158/2767-9764.CRC-23-0276)
Supplement: Supplementary Figure S2 — shows efficient knock-down of BAP1 via inducible shRNA in uveal melanoma. [file crc-23-0276-s02.pdf]

Supplementary Figure S2

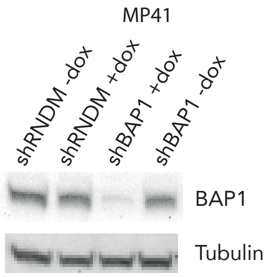

Supplementary Figure S2. Inducible BAP1 knock-down in uveal melanoma cell line MP41. Validation of BAP1 knock-down upon induction of the shRNA against BAP1 with doxycycline, both induced and uninduced controls are shown.
