# Supplementary material for: Combined Inhibition of EZH2 and FGFR is Synergistic in BAP1-deficient Malignant Mesothelioma
Source: Cancer Res Commun. 2024 Jan 3;4(1):18–27. doi: 10.1158/2767-9764.CRC-23-0276 (PMC10763530; doi:10.1158/2767-9764.CRC-23-0276)
Supplement: Supplementary Figure S3 — shows stable body weight of mice treated with the combination. [file crc-23-0276-s03.pdf]

Supplementary Figure S3

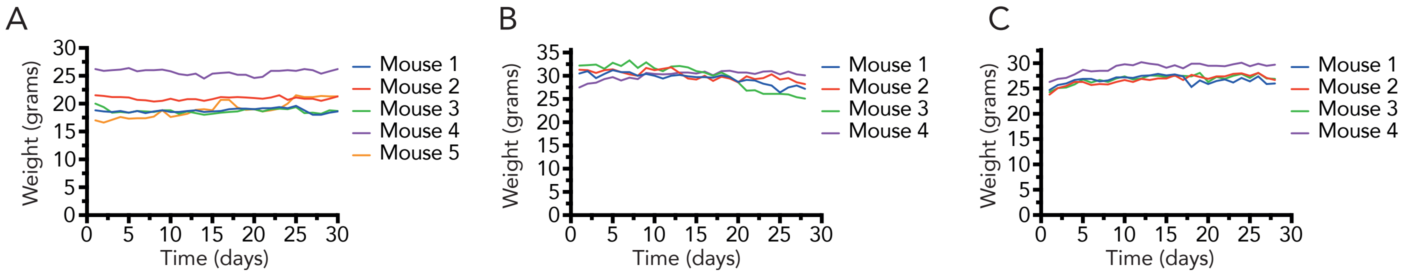

Supplementary Figure S3. Body weight of mice treated with EZH2 inhibition together with FGFR inhibition. A, Body weight of individual mice in the treatment group over time. Shown are C57BL/6 mice from the AZD4547/Tazemetostat cohort. **B,** Body weight of individual mice in the treatment group over time. Shown are NOD-Scid IL2Rγnull mice from the AZD4547/GSK126 cohort. **C,** Body weight of individual mice in the treatment group over time. Shown are NOD-Scid IL2Rγnull mice from the AZD4547/Tazemetostat cohort.
